# Annotations

**Published:** 1903-01-17

**Authors:** 


### ANNOTATIONS.

### The Imperial Vaccination League.

THE reports made by the three sub-committees appointed by the Imperial Vaccination League to investigate certain points connected with vaccination, are in favour (*a*) of compulsory revaccination at the age of twelve, and revaccination of all small-pox contacts; (*b*) of transferring the administration of the vaccination laws from the Poor Law to some other authority charged with public health functions; and (*c*) of supplying Government lymph to all qualified practitioners who may desire to use it. In regard to the questions concerning the use of glycerinated calf lymph the committee say that the present conditions under which this lymph is prepared and sold by private makers and vendors are unsatisfactory, and that all establishments for the preparation of calf lymph in England should be under Government inspection and control. This is but right and reasonable. We have again and again pointed out how objectionable is the present system which, although it might be put up with were vaccination a merely private affair which each person might accept or decline, and as to which, if accepted, he would take the whole responsibility, is absolutely intolerable when it is done under compulsion. We know no more striking instance of confidence in the good faith and honesty of a Government than that at its bidding a mother should offer her own child to be inoculated, with what after all is a disease, for the good of the community. Such trustful confidence surely throws upon the Government the obligation of doing all that is possible to secure the purity of the lymph which is used, not by public vaccinators alone, but by all who are allowed to undertake the work. Nothing is more surprising than the contrast between the official eagerness to get vaccination done and the official indifference as to how it is done and what kind of lymph is used for the purpose.

### Typhoid Fever Infection.

WE ought to regard with much jealousy anything which tends to throw doubt upon the importance of every community taking the greatest pains to secure the purity of its water supply. Hence we must refer in terms of considerable disapproval to the suggestion which has lately been made by Professor Koch to the effect that in the prevention of typhoid fever special precautions with regard to drinking water may be dispensed with provided sufficient care be taken to ensure the isolation of every individual suffering from the disease, and the complete disinfection of his clothes and excreta. If it were a matter of mere academic argument it might, indeed, be readily conceded that if the circuit of the infective bacillus from man to man be once broken the effect will be to stop the infection, and that it really does not much matter at which particular link in the chain the rupture takes place. But when it is proposed to put a stop to typhoid fever by the isolation of every case of the disease and the destruction of every particle of infection, we are met at once by two difficulties, that of the undetected case and that of the convalescent who still discharges germs. That these are real difficulties one need not hesitate to admit, especially when we bear in mind the amount of movement of population which is constantly taking place. By ensuring a pure water supply we can without doubt so far protect a whole community that the accidental presence of an infected stranger ceases to be a source of danger. If, however, leaving the water supply alone, we trust to the isolation and disinfection of those who are known to be affected, a single visit from a patient in the ambulant stage of fever might infect the whole community. We readily accept Professor Koch's statement that, by completely isolating the sick, he has been able to stamp out typhoid fever from a group of villages which had been notorious for the frequency of its typhoid cases. Perhaps, in a much-governed country like Germany, in which every new arrival must give an account of himself, there may be more chance of such an experiment succeeding than we are aware of. But in such a country as England, a land of freedom and of tramps, it is only by securing the purity of the water that we can secure anything approaching safety from typhoid infection. We need hardly say, however, that every care should be taken—far more care than usually is taken—to disinfect the discharges from all known cases of typhoid fever.

### Professor Lorenz and his Method.

THE visit of Professor Lorenz of Vienna to this country, and the demonstrations which he has been giving of his method of treating congenital dislocation of the hip draw renewed attention to this interesting condition. The main peculiarity of Lorenz's method is that, eschewing all incision and attempts to divide the capsule of the joint or the contracted tendons, he deals with the case by what we may term a scientific system of "bone setting." By careful, but in many cases very forcible, manipulation, the femur being made to move in every direction possible in the normal condition, by working the thigh up and down, by very forcibly abducting it, by hyperextending it, and by rotating it on its axis, all adhesions are so broken down that the limb lies almost flaccid in the surgeon's hands. The reposition of the head, or its remains, into such an acetabulum as exists is then accomplished, and the limb is fixed in a strongly abducted and somewhat everted position in plaster of Paris. Finally, the patient is encouraged to walk, it being found that in this way the re-formation of the joint is encouraged. In many cases the results have been brilliant, but it must not be imagined that the proceeding is altogether free from danger. It is spoken of as a bloodless method, but although there is no external hæmorrhage, there is often a very considerable internal effusion of blood into and around the lacerated structures. Even in Lorenz's own hands one case of gangrene of the thigh, one death from chloroform, two from combined shock and chloroform, and eleven fractures of the femur have been reported (*Annals of Surgery*, August, 1902), besides minor accidents. It is reported from New York that when operating lately in that city a death on the table was barely averted, and that in one of the patients treated by Professor Lorenz during his recent visit to America, fracture of the femur occurred, so that the suggestion which has been made that such accidents are due to the inexperience natural to the early days of a new method hardly holds true.

